# Lipocalin 2 Expression and Secretion Is Highly Regulated by Metabolic Stress, Cytokines, and Nutrients in Adipocytes

**DOI:** 10.1371/journal.pone.0096997

**Published:** 2014-05-12

**Authors:** Yuanyuan Zhang, Rocio Foncea, Jessica A. Deis, Hong Guo, David A. Bernlohr, Xiaoli Chen

**Affiliations:** 1 Department of Food Science and Nutrition, University of Minnesota-Twin Cities, Saint Paul, Minnesota, United States of America; 2 Department of Biochemistry, Molecular Biology and Biophysics, University of Minnesota-Twin Cities, Minneapolis, Minnesota, United States of America; Warren Alpert Medical School of Brown University, United States of America

## Abstract

Lipocalin 2 (Lcn2) has been recently characterized as a new adipokine having a role in innate immunity and energy metabolism. Nonetheless, the metabolic regulation of Lcn2 production in adipocytes has not been comprehensively studied. To better understand the Lcn2 biology, we investigated the regulation of Lcn2 expression in adipose tissue in response to metabolic stress in mice as well as the control of Lcn2 expression and secretion by cytokines and nutrients in 3T3-L1 adipocytes. Our results showed that the mRNA expression of Lcn2 was upregulated in white and brown adipose tissues as well as liver during fasting and cold stress in mice. Among pro-inflammatory cytokines TNFα, IL-1β, and IL-6, IL-1β showed most profound effect on Lcn2 expression and secretion in 3T3-L1 adipocytes. Insulin stimulated Lcn2 expression and secretion in a dose-dependent manner; this insulin effect was significantly abolished in the presence of low concentration of glucose. Moreover, insulin-stimulated Lcn2 expression and secretion was also attenuated when glucose was replaced by 3-O-methyl-d-glucose or by blocking NFκB pathway activation. Additionally, we showed that palmitate and oleate induced Lcn2 expression and secretion more significantly than EPA, while phytanic acid reduced Lcn2 production. Our results demonstrated that Lcn2 production in adipocytes is highly responsive to metabolic stress, cytokines, and nutrient signals, suggesting an important role of Lcn2 in adipocyte metabolism and inflammation.

## Introduction

Lipocalin 2 (Lcn2) was initially identified as a secreted protein from human neutraphils [1], [2]. Belonging to the same lipocalin superfamily members of fatty acid binding proteins and retinol binding proteins, Lcn2 also possesses a lipid binding domain, capable of binding small hydrophobic molecules [3]. Lcn2 is expressed in multiple tissues including uterus, bone marrow, immune cells, liver, spleen, and kidney in mice [4]–[6]. Recently, our group and others have discovered that Lcn2 is abundantly expressed and secreted by adipocytes. Since then, the role of Lcn2 as a new adipokine in metabolism has been explored [7], [8].

The promoter region of Lcn2 contains the binding sites of nuclear factor-κB (NFκB) and CCAAT enhancer binding protein (C/EBP) [9] as well as several nuclear receptor response elements including glucocorticoid response element [6], retinoic acid receptor response element [9], and estrogen response element [10]. Recently, Zhao at al identified STAT1 binding sites in the Lcn2 promoter, which mediates IFNγ induction of Lcn2 expression [11]. All the information indicates that the expression of Lcn2 is controlled by inflammation and metabolic conditions. Indeed, many pro-inflammatory and anti-inflammatory cytokines and factors such as lipopolysaccharide (LPS),tumor necrosis factor α (TNFα), interleukin-1β, interleukin-17, H_2_O_2_, dexamethasone, and retinoid acid have been reported to induce Lcn2 gene expression in a variety of cell types including immune cells, epithelial cells, hepatocytes and other types of cells[2], [6], [9], [12]–[15].

However, most of previous studies focus on the regulation of Lcn2 expression in non-adipocytes by inflammatory and oxidative stress inducers; the metabolic regulation of Lcn2 expression in adipocytes has not been well studied. Our group and others have reported that Lcn2 expression in adipose tissue is upregulated in genetic and diet-induced obese rodents [7], [8]. Studies with Lcn2 knockout mice have demonstrated that Lcn2 plays a significant role in obesity and insulin resistance [16], brown adipose tissue activation and thermogenesis [16]. Given the key role of Lcn2 in energy metabolism and obesity, it is of great importance to understand what factors regulate Lcn2 expression and secretion in adipocytes. In this study, we investigated the expression and secretion of Lcn2 in adipocytes in response to metabolic stress, inflammatory stimulation, and nutrient signals. We found that Lcn2 expression in adipose tissue and liver was significantly upregulated by fasting and cold stress in mice. In adipocytes, Lcn2 expression and secretion can be stimulated by TNFα, IL-1β, or IL-6 after 24 h treatment. Among these three cytokines, IL-1β is the most potent inducer of Lcn2 production. Insulin is also shown to stimulate Lcn2 expression and secretion; this effect of insulin is dependent on glucose metabolism and NFκB signaling activation. Additionally, we found that fatty acids palmitate and oleate are the potent inducers of Lcn2 expression and secretion in adipocytes, while Eicospentaenoic acid (EPA) has a minimal effect. All the data demonstrate that Lcn2 production in adipocytes is highly regulated by metabolic stress, inflammatory and nutrient signals, suggesting an important role of Lcn2 in adipocyte metabolism and inflammation.

## Materials and Methods

### Animal studies

Male C57BL/6 mice were obtained from the Jackson Laboratory (Bar Harbor, ME). Mice were maintained at 22°C on a 12∶12 h light-dark cycle. Animal handling followed National Institutes of Health guidelines, and experimental procedures were approved by the University of Minnesota Animal Care and Use Committee. In the fasting study, mice on a regular chow diet at 12 weeks of age were fasted for 24 h or 48 hours and sacrificed.In the cold adaptive study, mice on the regular chow diet were exposed to 22°C or 4°C for 4 hours, with free access to water. After cold exposure, the mice were sacrificed immediately; tissue samples were collected for various assessments.

### Cell Cultures

3T3-L1 cells were cultured in DMEM with 10% bovine calf serum (Sigma Aldrich, Saint Louis, MO) and 100 IU/ml penicillin/streptomycin (Invitrogen, Carlsbad, CA) until confluence. Two days after confluence, the cells were induced for differentiation with the differentiation cocktail containing 10% fetal bovine serum (JRH Biosciences, Inc., Lenexa, KS), 115 µg/ml methylisobutylxanthine (Sigma Aldrich, Saint Louis, MO), 1 µg/ml insulin (Sigma Aldrich, Saint Louis, MO), and 390 ng/ml dexamethasone (Sigma Aldrich, Saint Louis, MO). The differentiation cocktail was replaced with DMEM containing 10% fetal bovine serum, 100 IU/ml penicillin/streptomycin and 1 µg/ml insulin two days later. The cultures were continued for additional 6 days.On day 7 of differentiation, adipocytes were starved in DMEM with 1 mg/ml glucose and 0.5% bovine calf serum for 12 hours, followed by various treatments as described in the figure legends. Both conditioned media and cells were collected for protein or RNA detection.

### Quantitative real-time RT-PCR

Total RNAs were extracted from mouse tissues or 3T3-L1 adipocytes using TRIzol reagent (Invitrogen, Carlsbad, CA) according to the manufacturer's instructions. After treatment with RQ1 DNase (Promega, Madison, WI), 1 µg RNA were used to synthesize the cDNA in reverse transcription with 5 µM oligo dT primer, 0.5 mM dNTP, 20 units RNasin Ribonuclease Inhibitor (Promega, Madison, WI), 5 mM DTT, and 100 units SuperScript II Reverse Transcriptase (Invitrogen, Carlsbad, CA). Real-time-PCR was performed using SYBR GreenER qPCR SuperMix Universal kit (Invitrogen Carlsbad, CA) with an ABI 7500 Real Time PCR System (Applied Biosystems, Foster City, CA). Results were analyzed using the software supplied with the 7500 system and presented as levels of expression relative to that of controls after normalizing to β-actin or GAPDH using ΔΔCt method. Statistical significance was determined by two-tailed Student's *t* test. Primer sequences for Lcn2 were 5′- TGAAGGAACGTTTCACCCGCTTTG -3′ and 5′- ACAGGAAAGATGGAGTGGCAGACA -3′.

### Western-blotting

Tissue samples were homogenized and solubilized in RIPA buffer (Sigma, St. Louis, MO). The lysates were centrifuged at 12,000 g for 10 minutes, and supernatants were collected. Lysates of 3T3-L1 adipocytes were prepared in a lysis buffer containing 25 mM Tris-HCl pH 7.5, 0.5 mM EDTA, 25 mM sodium chloride, 10 mM sodium fluoride, 1 mM sodium vanadate, 1% Nonidet P-40 and protease inhibitor cocktails (Diagnostic Roche, Branchburg, NJ). Protein concentrations of lysates were detected with bicinchoninic acid method (Pierce, Rockford, IL). Equivalent proteins or same volume of conditioned media were loaded and separated on SDS-PAGE and then electro-transferred to nitrocellulose membranes. Membranes were incubated with anti-LCN2 (R&D System, Minneapolis, MN), anti-Phospho-NFκB p65 (Ser536) and anti-Phospho-STAT3 (Tyr705) (Cell Signaling, Danvers, MA) and anti-actin antibodies after blocking with TTBS (20 mM Tris-HCl pH 7.5, 0.5 M NaCl, 0.1% Tween-20) containing 5% milk (Fisher Scientific, Pittsburgh, PA). The membranes were then washed in TTBS and incubated with corresponding secondary antibodies conjugated to horseradish peroxidase (GE Healthcare Bio-Sciences Corp., Piscataway, NJ). The signals were detected by ECL plus Western Blotting Detection Reagents (GE Healthcare Bio-Sciences Corp., Piscataway, NJ).

## Results

### Regulation of Lcn2 expression in adipose tissue during metabolic stress

**Figure 1:**
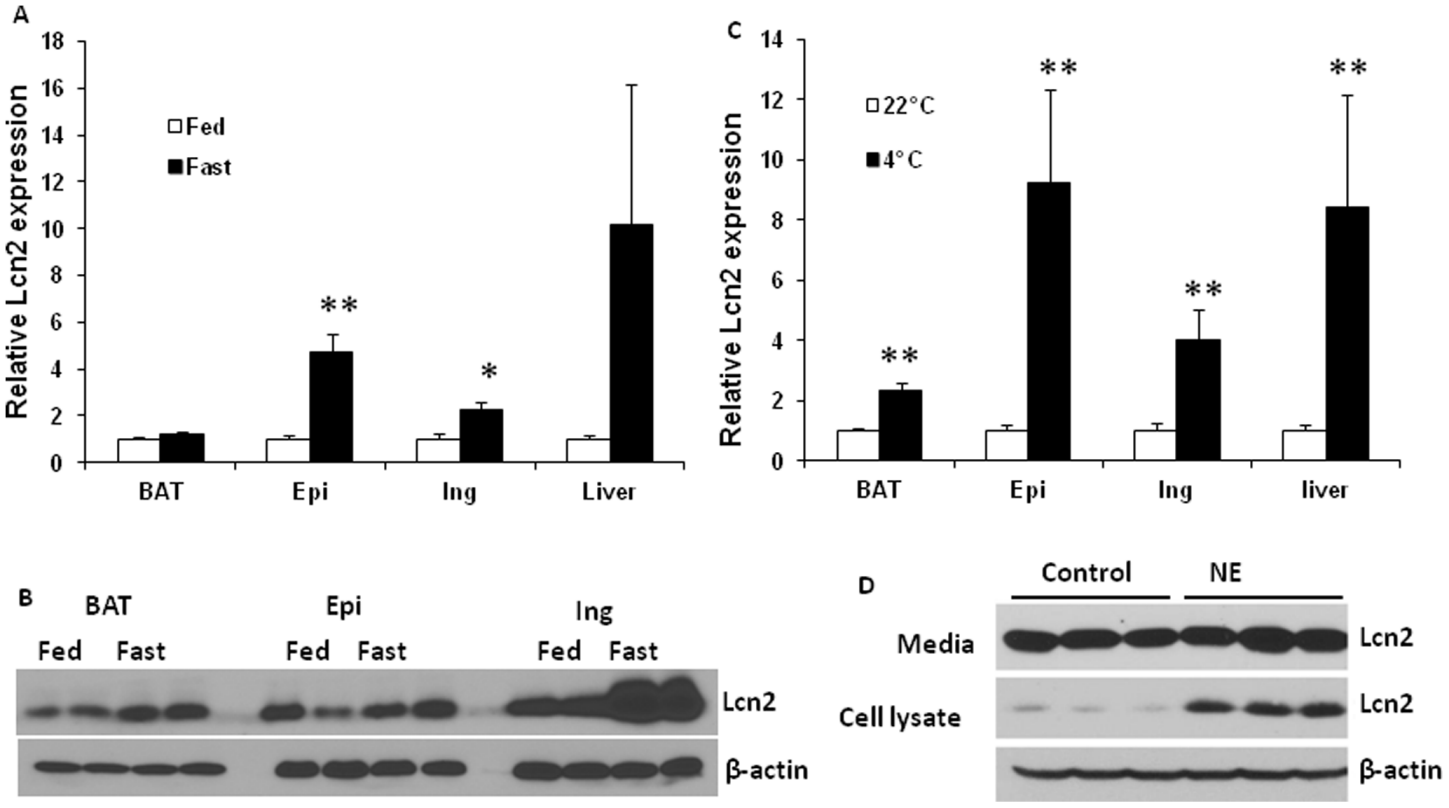
The Lcn2 expression in adipose tissue depots and liver during metabolic stress. (A) the mRNA expression of Lcn2 in brown adipose tissue (BAT), epididymal adipose tissue (Epi), inguinal adipose tissue (Ing), and liver in C57/BL6 mice at 12 weeks of age during 48 h fasting. (B) Lcn2 protein expression in BAT, Epi and Ing adipose tissue in C57/BL6 mice at 12 weeks of age during 24 h fasting. (C) the mRNA expression of Lcn2 in BAT, Epi and Ing adipose tissue, and liver in C57/BL6 mice at 12 weeks of age after exposed to 22°C or 4°C for 4 h. (D) Norepinephrine induces Lcn2 expression and secretion in 3T3-L1 adipocytes. 3T3-L1 cells on day 7 of differentiation were treated with or without 1 µM norepinephrine (NE) for 24 h. Conditioned media and cells were collected and subjected to immune-blotting with the antibody against Lcn2. The mRNA expression levels in fasted and cold-adapted mice were normalized to the levels in control mice and shown as fold changes. The results are presented as mean ± SE and represent two independent experiments (n = 4–6 in each experiment). * p<0.05, ** p<0.01; * Comparison between control and fasted or cold-exposed mice.

We and others have previously reported that Lcn2 is abundantly expressed in adipose tissue and shows upregualtion in obesity [7], [8]. Nonetheless, there has been no attempt to compare the metabolic regulation of Lcn2 expression in different depots of adipose tissue. Herein we investigated how Lcn2 expression in different fat depots is regulated during metabolic stress. First, we determined the effect of fasting on Lcn2 expression in adipose depots. In response to 48 h fasting, the mRNA expression of Lcn2 was significantly increased in inguinal and epididymal adipose tissue as well as liver (Fig 1A). Consistent with Lcn2 gene expression, Lcn2 protein expression was also increased in white and brown adipose tissue after 24 h fasting; this increase is most profound in inguinal depot (Fig. 1B). Second, we looked at the Lcn2 expression in response to cold stress. Male mice at 12 weeks of age were exposed to 22°C or 4°C for 4 h. The results showed that Lcn2 gene expression was significantly upregulated in all adipose tissue depots examined and liver (Fig 1C). Lastly, we treated 3T3-L1 adipocytes with 1 µM norepinephrine (NE), an agonist of β3-adrenergic receptor, to examine if adrenergic stimulation mediates the upregulation of Lcn2 expression in adipose tissue during fasting and cold stress. As shown in Fig 1D, NE treatment for 24 h led to a marked increase in intracellular Lcn2 protein, but a slight increase in Lcn2 secretion.

### Regulation of Lcn2 expression and secretion by cytokines in adipocytes

**Figure 2:**
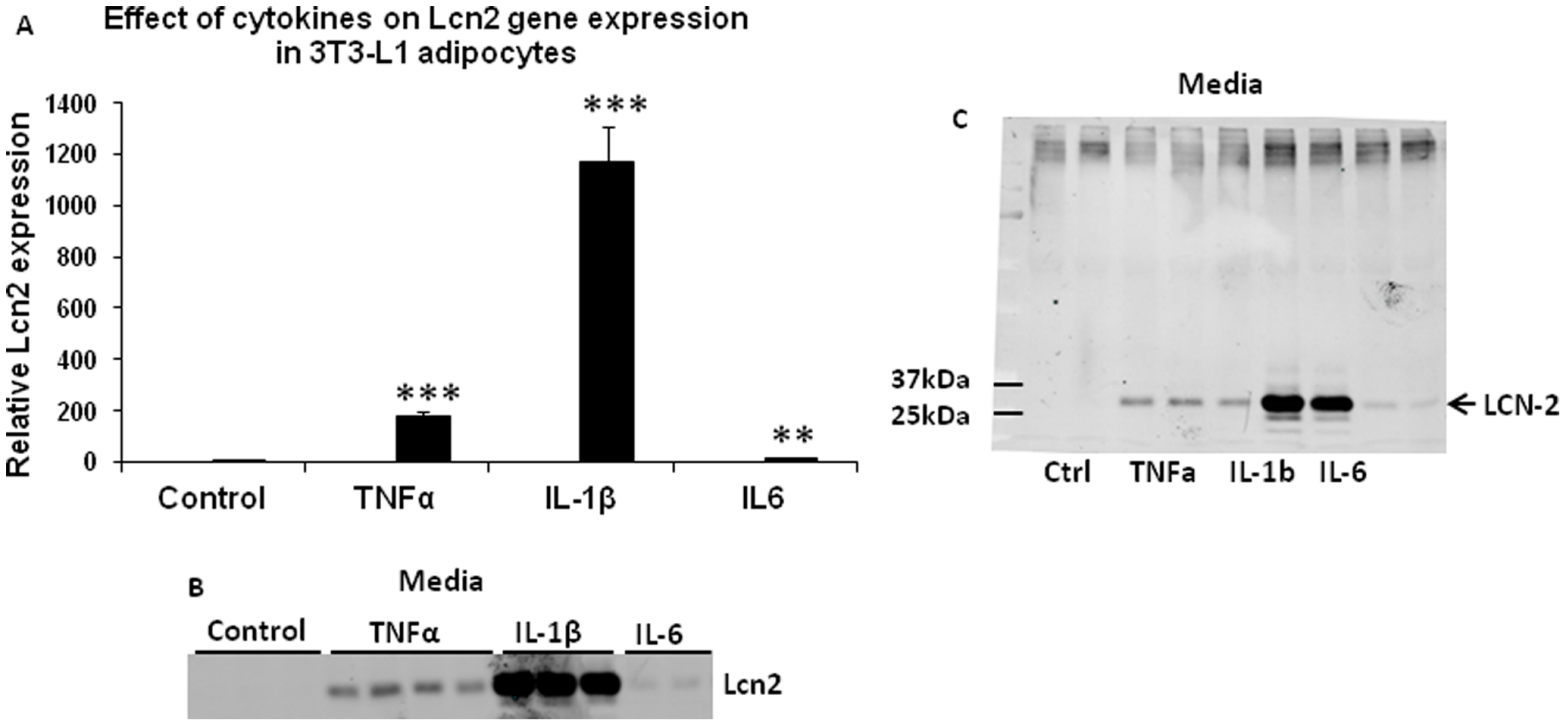
Regulation of Lcn2 mRNA expression and secretion by cytokines in adipocytes. (A) the mRNA expression of Lcn2 in 3T3-L1 adipocytes treated with TNFα, IL-1β, or IL-6 at the concentration of 1 nM for 16 h. The mRNA expression levels in cytokine-treated adipocytes were normalized to the levels in control adipocytes and shown as fold changes. The results are presented as mean ± SE and represent two independent experiments. ** p<0.01, *** p<0.001; * Comparison between control and treated cells. (B) Lcn2 secretion in 3T3-L1 adipocytes treated with TNFα, IL-1β, or IL-6 at the concentration of 1 nM for 24 h. Conditioned media were collected and subjected to immune-blotting with the antibody against Lcn2. The results represent two independent experiments. (C) Western-blotting of Lcn2 in 3T3-L1 adipocytes treated with cytokines under the non-reducing condition.

Previous studies have shown that Lcn2 promoter region contains NFκB binding sites [9]; TNFα and LPS are the two strong inducers of Lcn2 expression in human neutrophils[2]. Herein, we examined the effect of cytokines including TNFα, IL-1β, and IL-6 on Lcn2 expression and secretion in adipocytes. As illustrated in Fig. 2A, all three cytokines significantly upregulated the mRNA expression of Lcn2 in 3T3-L1 adipocytes after 24 h treatment. Interestingly, three cytokines at the same concentration (1 nM) displayed differential capability of stimulating Lcn2 gene expression in adipocytes. Among three cytokines, IL-1β is the strongest inducer of Lcn2 gene expression, while IL-6 had the weakest effect. Consistent with the effect of three cytokines on Lcn2 mRNA expression, IL-1β showed the most profound effect on Lcn2 secretion compared to TNFα and IL-6 (Fig 2B). Additionally, Western-blotting of Lcn2 in culture media performed under the non-reducing condition showed that there was only a single band at ∼25 kDa that was observed; no higher molecular weight of Lcn2 was detected.

### Regulation of Lcn2 expression and secretion by glucose and insulin in adipocytes

**Figure 3:**
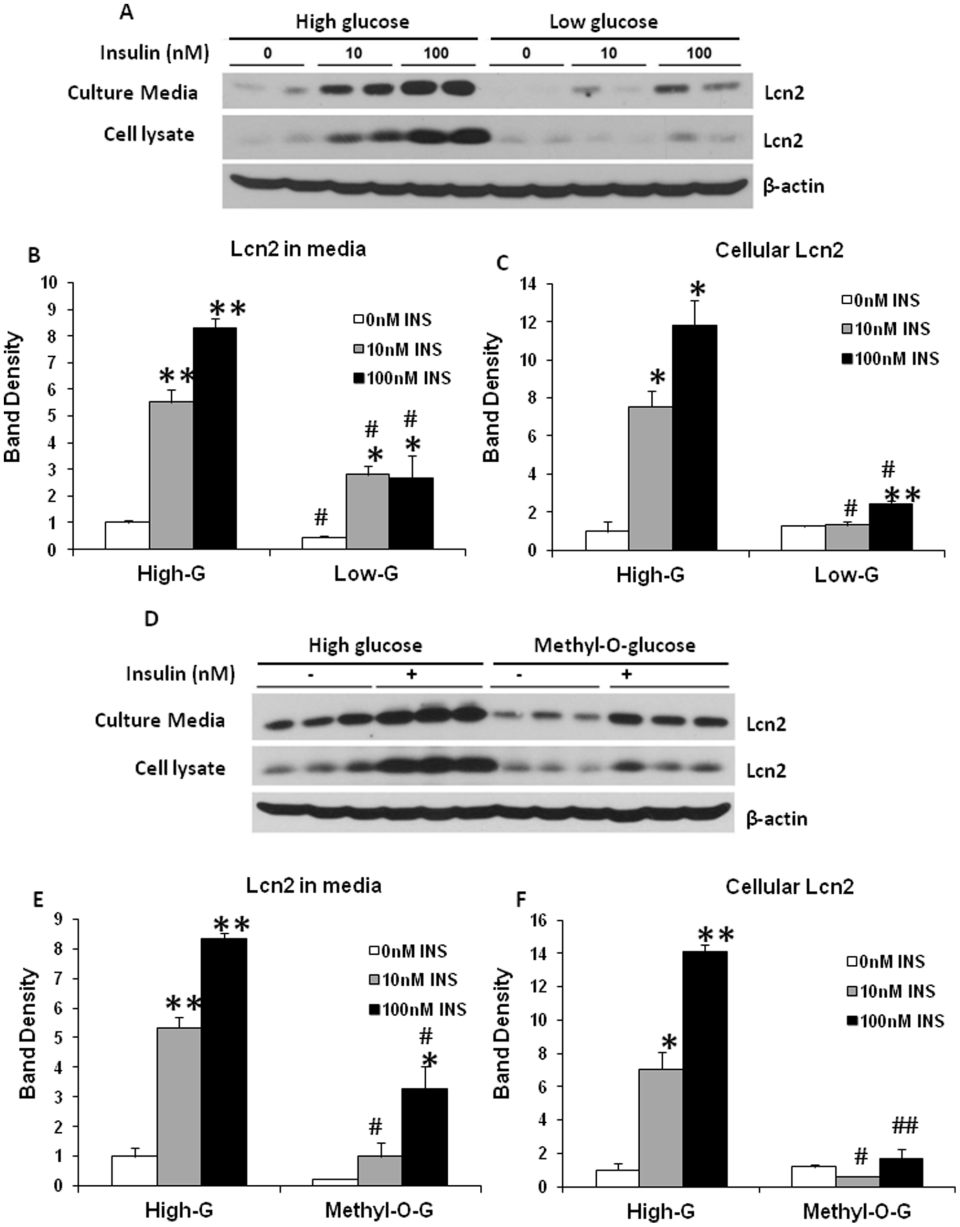
Regulation of Lcn2 expression and secretion by insulin and glucose in 3T3-L1 adipocytes. (A) Dose-dependent effect of insulin on Lcn2 expression and secretion in the presence of high or low concentration of glucose. On day 7 of differentiation, cells were either maintained in DMEM containing high concentration of glucose (4.5 g/L) or switched to DMEM containing low concentration of glucose (1 g/L). Insulin was added at different concentrations as indicated. After 24 h, conditioned media and cells were collected and subjected to western-blotting with the antibody against Lcn2. (B and C) the quantification of band density shown in A. The results were presented as mean ± SE. (D) Effect of 3-O-methyl-d-glucose (Methyl-O-G) on insulin-stimulated Lcn2 expression and secretion. On day 7 of differentiation, cells were changed to high-glucose DMEM containing 4.5 g/L glucose or DMEM containing 1 g/L glucose and 3.5 g/L 3-O-methyl-d-glucose. Insulin was added at different concentrations as indicated. After 24 h, conditioned media and cells were collected and subjected to western-blotting. (E and F) the quantification of band density shown in D. The results were presented as mean ± SE.*or # p<0.05, ** p<0.01. *Comparison between control and insulin treatments. # Comparison between low and high glucose treatment or between Methyl-O-G and high glucose treatment.

As shown in our previous studies, Lcn2 expression is increased in adipose tissue in obesity. Chronic low-grade inflammation in adipose tissue is well known as one of the characteristics in diet-induced obesity. Metabolic stress such as nutrient overload is believed to be a metabolic insult that triggers low-grade inflammation in obese adipose tissue. To understand better the role of Lcn2 in adipose tissue inflammation and metabolism, it would be of importance to examine if Lcn2 expression is regulated by nutrients. In this experiment, we investigated how glucose and insulin regulate Lcn2 expression and secretion in adipocytes. Fully-differentiated 3T3-L1 adipocytes were treated with or without 100 nM insulin in DMEM containing either high glucose (4.5 g/L glucose) or low glucose (1 g/L glucose) for 24 hours. In the absence of insulin, intracellular Lcn2 protein levels were not significantly different between adipocytes cultured in low-glucose and those in high-glucose DMEM (Fig 3A, 3C, 3D, and 3F), while Lcn2 secretion was significantly higher in adipocytes cultured in high-glucose DMEM compared to those in low-glucose DMEM (Fig. 3A, 3B, 3D, 3E). The addition of insulin led to a marked increase in Lcn2 expression and secretion in a dose-dependent manner (Fig 3A–3C). Interestingly, the effect of insulin was almost diminished in adipocytes cultured in the condition of low glucose (Fig 3A–3C). Considering that insulin stimulates glucose uptake into adipocytes, we then addressed the question of whether the effect of insulin on Lcn2 expression depends on glucose uptake and metabolism. Hence we substituted glucose with the same concentration of 3-O-methyl-d-glucose, a poorly metabolized analog of glucose, and performed the similar experiment. Interestingly, we found that the induction of Lcn2 expression by insulin was markedly attenuated in DMEM containing 3-O-methyl-d-glucose (Fig. 3D–3F). Our results suggest that glucose metabolism is required for the insulin induction of Lcn2 expression and secretion in adipocytes.

### Blocking NFκB signaling pathway reduces insulin-stimulated Lcn2 secretion in adipocytes

**Figure 4:**
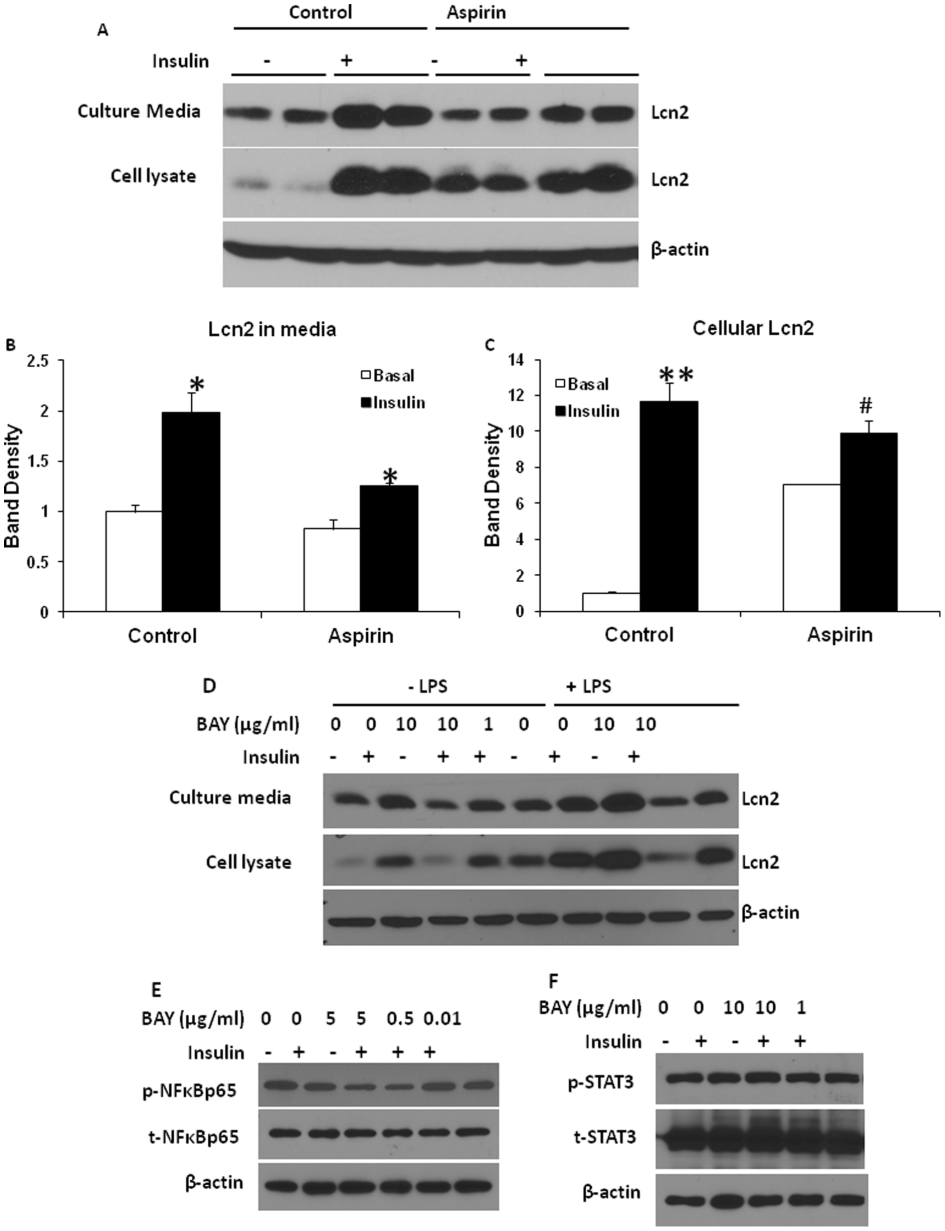
Effect of blocking NFκB signaling pathway activation on insulin and glucose induction of Lcn2 expression and secretion in adipocytes. (A) 3T3-L1 adipocytes on day 7 of differentiation were treated with 5 mM aspirin in high-glucose DMEM with 100 nM insulin for 24 h. Conditioned media and cells were collected and subjected to western-blotting with the antibody against Lcn2. The quantification of band density shown in B and C. The results were presented as mean ± SE.*or # p<0.05, ** p<0.01. *Comparison between control and insulin treatment. # Comparison between control and Aspirin treatment. (D–F) 3T3-L1 adipocytes on day 7 of differentiation were pre-treated with various concentrations of BAY 11-7082 for 1 h followed by the treatment of 100 nM insulin for 24 h in the presence or absence of LPS. Conditioned media and cells were collected and subjected to western-blotting with the antibody against indicated proteins.

High glucose is known to induce inflammation and activate NFκB pathway in endothelial cells [17]–[19] and vascular smooth muscle cells [20], [21]. Our results have shown above that insulin stimulation of Lcn2 expression and secretion is dependent on glucose uptake and metabolism. To test if the activation of NFκB pathway is important for insulin-stimulated Lcn2 expression and secretion, we evaluated the consequences of blocking the transduction of NFκB signaling pathway on Lcn2 expression and secretion induced by insulin and glucose. Aspirin was used to inhibit the activation of NFκB signaling pathway. Salicylic acid, the main metabolite of aspirin, has been shown to modulate NF-κB pathway activation [22]. Our results demonstrated that the treatment of 5 mM aspirin for 24 hours significantly attenuated insulin-stimulated Lcn2 secretion in the presence of high concentration of glucose, but increased basal intracellular Lcn2 expression (Fig 4A–4C), suggesting that the effect of insulin and glucose on Lcn2 secretion is likely mediated by the inflammatory pathway. Additionally, we used BAY 11-7082, a different inhibitor of NFκB activation to further test the mediation of NFκB signaling pathway in insulin-stimulated Lcn2 expression and secretion. Similar to aspirin, BAY 11-7082 reduced the insulin stimulation of Lcn2 secretion (Fig 4D). Moreover, we found that lipopolysaccharides (LPS), a strong activator of NFκB was more potent in stimulating Lcn2 expression and secretion in adipocytes compared to insulin (Fig 4D). More interestingly, PLS and insulin had an additive effect on Lcn2 production, and the effects of LPS alone or insulin and LPS in combination were significantly blocked by BAY 11-7082 (Fig 4D). Fig 4E and 4F confirmed that the phosphorylation of NF-κB p65, but not STAT3 was reduced by BAY 11-7082.

### Regulation of Lcn2 expression and secretion by fatty acids in adipocytes

**Figure 5:**
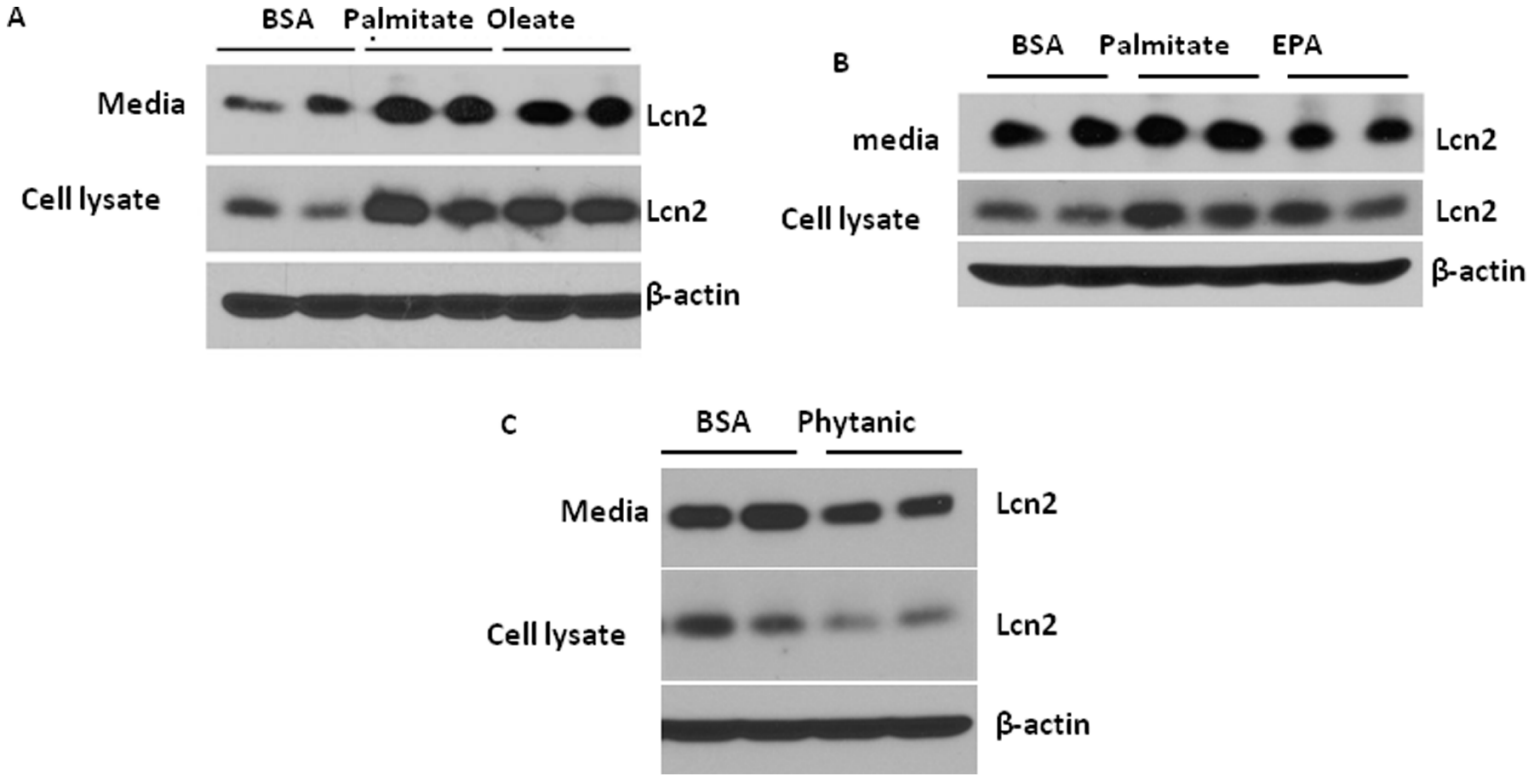
Effect of fatty acids on Lcn2 expression and secretion in adipocytes. 3T3-L1 adipocytes on day 7 of differentiation were treated with 100 µM BSA with or without (A) 250 µM palmitate, 400 µM oleate, (B) 400 µM EPA, or (C) 10 µM phytanic acid in low-glucose media in the presence of insulin. After 24 h, conditional media and cells were collected and subjected to western-blotting with the antibody against Lcn2. The results represent for two independent experiments.

In addition to glucose, fatty acids are the nutrient signals that play an important role in mediating nutrient overload-induced inflammation in adipose tissue. We next determined how different types of fatty acids regulate Lcn2 expression and secretion in adipocytes. Differentiated 3T3-L1 adipocytes were treated with 250 µM palmitate (C16∶0), 400 µM oleate (C18∶1), or 400 µM eicosapentaenoic acid (EPA C20∶5) in the presence of 100 µM fatty acid-free BSA and 100 nM insulin for 24 hours. We found that intracellular and secreted Lcn2 was markedly increased after 24 h-treatment of palmitate and oleate at a similar extent (Fig. 5A and 5B). Compared with palmitate, EPA had less stimulatory effect on Lcn2 expression and secretion (Fig. 5B). Phytanic acid is a branched chain fatty acid that is metabolized in the peroxisome. However, treatment of 10 µM phytanic acid for 24 h led to a reduction in intracellular and secreted Lcn2 levels in adipocytes (Fig. 5C).

## Discussion

Accumulating evidence has linked Lcn2 to obesity, insulin resistance, inflammation, and metabolic diseases [7], [8], [16], [23]–[27]. However, the important information regarding the regulation of Lcn2 production in adipocytes, especially in response to metabolic stress and nutrients is currently lacking. In this study, we investigated how metabolic stress, inflammatory and nutrient signals regulate LCN2 expression and secretion in adipocytes. We demonstrate that Lcn2 gene expression is upregulated in brown and white adipose depots of male mice during fasting and cold stress. Additionally, we show that Lcn2 protein expression and secretion is regulated by cytokines, insulin, glucose, and fatty acids in adipocytes.

In order to better understand how Lcn2 plays a role in energy metabolism, we determined if and how Lcn2 expression in adipose depots is regulated during fasting and cold stress. Our results showed that Lcn2 expression in BAT and WATs was upregulated in response to fasting, and this response seems to be depot-different. For instance, inguinal depot and BAT have a more significant increase in Lcn2 protein expression than epididymal depot during fasting. We also showed that the gene expression of Lcn2 was upregulated by cold stimulation in all adipose tissues examined, including BAT, epididymal and inguinal adipose depots as well as liver. The results from 3T3-L1 adipocyte cultures demonstrated that Lcn2 production is directly stimulated by NE, suggesting that increased NE levels during cold stress may be responsible in part for cold-induced upregulation of Lcn2 expression in adipose tissues *in vivo*. As we shown in the results, Lcn2 expression and secretion is also stimulated by insulin, glucose, and fatty acids. Since insulin and glucose levels are reduced, while fatty acid and NE levels are increased during fasting, it is likely that elevated levels of fatty acids and NE play a major role in stimulating Lcn2 expression and secretion during fasting.

Lcn2 has been known to play a key role in innate immunity and protect against bacterial infection [28], [29]. Previous studies have demonstrated that LPS and cytokines are the inducers of Lcn2 expression in immune cells. In this study, we found that TNFα, IL-1β, and IL-6 are also inducers of Lcn2 production in adipocytes. Interestingly, these cytokines with the same concentration have a differential capability of stimulating Lcn2 expression and secretion in adipocytes albeit they are all able to significantly increase Lcn2 gene expression and secretion. IL-1β is the most potent inducer, while IL-6 has a weakest stimulatory effect. Moreover, we show that Lcn2 is secreted into the culture media as a monomer, but does not form multimeric complex in response to cytokine stimulation. Lcn2 promoter region contains NFκB and STAT1 binding sites which primarily mediate the induction of Lcn2 transcription by cytokines. TNFα is known to activate NFκB pathway for its inflammatory effect. A recent study suggests that IFγ-1 stimulates Lcn2 expression via activating STAT1, but is less potent than TNFα [11]. However, IFγ-1and TNFα together have a significant additive effect on Lcn2 expression in adipocytes [11], suggesting that they regulate Lcn2 transcription through a different activation pathway. Our results show that IL-1β stimulates much higher levels of Lcn2 expression than TNFα and IL-6 in adipocytes. We speculate that IL-1β effect on Lcn2 expression is via activating multiple signaling pathways that control Lcn2 transcription.

Different from infection-triggered inflammation, obesity-related adipose tissue inflammation is characterized as chronic low-grade inflammation. Metabolic insults are the main triggers of adipose tissue inflammation. It has been known that certain nutrients such as glucose and saturated fatty acids are naturally pro-inflammatory. To understand better the role of Lcn2 in obesity and adipose tissue inflammation, we examined the regulation of Lcn2 expression and secretion in adipocytes by nutrients. Our results show that insulin increases Lcn2 protein expression and secretion in 3T3-L1 adipocytes; this result is consistent with a previous study showing that insulin increases Lcn2 production in cultured human omental adipose tissue explants [30]. However, the role of glucose in the insulin-stimulated Lcn2 expression in adipose tissue was not investigated in the previous study. Interestingly, we found that the stimulatory effect of insulin on Lcn2 expression and secretion is glucose-dependent. Further, we demonstrate that insulin-stimulated Lcn2 expression and secretion was blunted by substituting glucose with 3-O-methyl-d-glucose, suggesting that glucose metabolism is required for the effect of insulin on Lcn2 expression. This data proves the hypothesis that insulin-stimulated glucose uptake and metabolism is the main trigger of Lcn2 transcription. Since insulin stimulates both Lcn2 expression and secretion at the similar degree, it is possible that insulin does not regulate the process of Lcn2 secretion. Increased Lcn2 in media is due to increased expression of Lcn2, suggesting that insulin and glucose regulate Lcn2 production primarily at the level of transcription. As described above, NFκB pathway activation is an important mediator of inflammation-induced Lcn2 expression. Therefore, it is reasonable to speculate that insulin and glucose induce Lcn2 expression through promoting glucose catabolism (oxidation) and ROS production thereby NFκB activation. Indeed, our results that the insulin stimulation of Lcn2 expression and secretion was attenuated when NFκB pathway activation was blocked by aspirin and BAY 11-7082 support the above hypothesis. Most importantly, our result is supported by recent studies [31], [32]. In the studies, Zhao et al provided direct evidence showing the binding of NF-κB to the human LCN2 promoter in human adipocytes, and the NF-κB signaling pathway is required for the TNFα-induced LCN2 expression [32]. Since STAT1 was also found to bind Lcn2 promoter [31], it is likely that STAT signaling pathway may also contribute to the mediation of insulin-stimulated Lcn2 production. Our results indicate that blocking NFκB activation only resulted in the partial inhibition of insulin-stimulated Lcn2 production. We also showed that BAY 11-7082 does not reduce STAT3 activation. Therefore, we believe that NFκB pathway is responsible only in part for insulin-induced Lcn2 production.

In addition, we looked at the effect of fatty acids (saturate fatty acid, monounsaturated fatty acid and polyunsaturated fatty acid) on Lcn2 expression and secretion in adipocytes. We found that palmitate and oleate are the stronger inducers of Lcn2 expression and secretion than EPA in 3T3-L1 adipocytes. However, phytanic acid, a branched fatty acid, significantly reduced Lcn2 protein expression and secretion after 24-hour treatment. Phytanic acid has several unique features. Firstly, unlike other types of fatty acids, phytanic acid cannot undergo β-oxidation in mitochondria due to the presence of the first methyl-group at the 3-position. Instead it is metabolized by α-oxidation in the peroxisome and converted into pristanic acid. Pristanic acid undergoes several rounds of β-oxidation to form medium chain fatty acids which are finally metabolized in mitochondria. Secondly, phytanic acid has been reported to enhance insulin-independent glucose uptake in primary rat hepatocytes [33], while other types of fatty acids inhibit glucose uptake [34]. Thirdly, phytanic acid and its metabolites have been reported to activate retinoid X receptor (RXR) [35] and PPARα [36], promoting peroxisomal and mitochondrial fatty acid oxidation [37], [38]. Lastly, phytanic acid has been indicated to be positively associated with energy expenditure as an efficient inducer of UCP1 expression and brown adipocyte differentiation [39], [40]. All of the above aspects, such as peroxisome activity, fatty acid oxidation, energy expenditure, may be associated with the unique regulatory effect of phytanic acid on Lcn2 expression.

In summary, we have demonstrated that Lcn2 expression and secretion in adipocytes is highly regulated by metabolic stress and nutrient signals. Fasting or cold stress increases *Lcn2* gene expression in BAT and WATs in mice. In 3T3-L1 adipocytes, Lcn2 protein expression and secretion is directly regulated by metabolic hormones and nutrient signals. NE stimulates Lcn2 protein expression and secretion. Moreover, insulin stimulates Lcn2 protein expression and secretion in the glucose- and NFκB-dependent manner. Additionally, TNFα, IL-1β, palmitate, and oleate, are all the strong inducers of Lcn2 expression and secretion. Further studies are needed on the detailed mechanism for the regulation of Lcn2 expression in adipocytes, which will help understand better the role of Lcn2 in diet-induced obesity and adipose tissue inflammation.
